# Nurse-led digital therapeutics for type 2 diabetes in routine care: a retrospective cohort study of adherence, glycemic outcomes, and care continuity

**DOI:** 10.3389/fpubh.2026.1889568

**Published:** 2026-08-12

**Authors:** Jinyan Ma, Lintao Zhen

**Affiliations:** 1 Department of Nephrology, West China Hospital, Sichuan University, Chengdu, Sichuan, China; 2 Sichuan City Vocational College, Chengdu, Sichuan, China

**Keywords:** adherence, diabetes nursing, digital therapeutics, eHealth literacy, real-world evidence, retrospective cohort study, self-management, type 2 diabetes

## Abstract

**Background:**

Digital therapeutics can extend diabetes self-management beyond clinic visits, but sustained engagement, safe interpretation of patient-generated data, and integration with routine care remain challenging.

**Objective:**

This study examined the real-world association between a nurse-led digital therapeutics pathway and clinical, behavioral, and patient-experience outcomes among adults with type 2 diabetes.

**Methods:**

De-identified routine-care records were used to compare a structured nurse-led digital therapeutics pathway with usual care over 24 weeks. Eligible baseline records were classified by documented routine-care pathway exposure, and outcome analyses used complete 24-week records. The nurse-led pathway included digital readiness assessment, onboarding education, adherence monitoring, tiered follow-up, feedback reports, and coordination with physicians and technical staff.

**Results:**

Complete 24-week data were available for 57 patients in the nurse-led pathway and 56 receiving usual care. Adherence was 78.3% and 54.7%, respectively (between-pathway difference, 23.6 percentage points; *p* < 0.001), while mean glycated hemoglobin change was −0.8% and −0.3% (difference, −0.5 percentage points; *p* < 0.001). The nurse-led pathway was also associated with higher PSQ-18 satisfaction scores, better self-management behavior, and lower glycemic variability. Pathway exposure remained the strongest factor associated with adherence after adjustment.

**Conclusion:**

In this single-center retrospective cohort, the nurse-led digital therapeutics pathway was associated with more sustained digital engagement and more favorable metabolic and patient-reported outcomes than usual care. Prospective multicenter studies are needed before causal or broad implementation claims can be made.

## 1 Introduction

Type 2 diabetes places a sustained self-management burden on patients, families, and health systems. Beyond pharmacological treatment, patients must monitor glucose, adjust diet and physical activity, recognize warning signs, and maintain long-term medication routines, often while balancing work, family responsibilities, and uneven access to professional support. The global prevalence of diabetes continues to rise, and many adults with type 2 diabetes do not achieve recommended glycemic targets despite effective therapeutic options (1–4). These gaps have encouraged wider use of mobile health tools, remote monitoring, and digital therapeutics, which can support recording, reminders, feedback, and behavioral reinforcement when they are appropriately connected to clinical care (5–7).

Randomized trials and systematic reviews indicate that digital tools can improve glycated hemoglobin and self-management for some adults with type 2 diabetes, although outcomes vary with intervention design, patient engagement, and access to human support (8–11). A persistent limitation is that technology alone does not ensure sustained use, equitable access, patient safety, or clinically meaningful benefit. App fatigue, low eHealth literacy, incomplete data entry, and delayed responses to abnormal glucose patterns can undermine implementation, particularly among patients who need the most support (12–15). The relevant clinical question is therefore not merely whether a diabetes application is available, but how it is embedded in a care pathway that patients can use safely and consistently.

Nursing theory and diabetes education evidence provide a practical basis for this pathway-level perspective. Orem’s self-care deficit theory supports assessment of a patient’s capacity to perform daily diabetes-related tasks, whereas the transtheoretical model helps nurses tailor education to readiness for behavior change (16, 17). Consensus work on self-care interventions and diabetes self-management education likewise emphasizes that information alone is seldom sufficient; many patients need structured professional support to sustain health behaviors over time (18, 19). Nurse-led diabetes self-management education has been associated with improvements in glycated hemoglobin and cardiovascular risk factors, yet studies of digital therapeutics often describe the application more fully than the nursing workflow that makes routine use feasible (20, 21).

This study aimed to evaluate the real-world association between a nurse-led digital therapeutics pathway and clinical, behavioral, and patient-experience outcomes among adults with type 2 diabetes over 24 weeks. The primary objective was to compare glycated hemoglobin change and digital therapeutics adherence between patients managed through the nurse-led pathway and those receiving usual care. The secondary descriptive objective was to characterize the operational components, professional roles, and nursing activities embedded in the nurse-led pathway. Secondary evaluative objectives were to examine between-pathway differences in patient satisfaction, self-management behavior, and glycemic variability and to describe the nursing workload documented for the nurse-led pathway. Because the analysis relied on retrospective routine-care records, all findings were interpreted as observational associations rather than evidence of causal effectiveness.

## 2 Materials and methods

### 2.1 Study design, setting, and theoretical framework

This retrospective observational cohort study used de-identified electronic health records from a 24-week observation period in the endocrinology department of a tertiary hospital. Reporting was guided by STROBE and by the RECORD extension for studies using routinely collected health data (22, 23). All data came from routine clinical care; no prospective allocation, experimental manipulation, or additional patient contact was introduced for research purposes. Blinding was not implemented because pathway exposure had already occurred in routine practice. The 24-week period covered two glycated hemoglobin testing cycles and allowed assessment of both DTx adherence and glycemic outcomes. Given the absence of randomization, the analyses were designed to describe between-pathway associations while acknowledging potential residual confounding.

The theoretical framework integrated self-care deficit theory, health behavior promotion, and the transtheoretical model. In routine practice, nurses used these perspectives to identify digital therapeutics-related self-care barriers, assess facilitators of digital engagement, and select education or follow-up strategies according to each patient’s readiness for behavior change. The framework also emphasized clinical accountability: data from the digital tool were treated as decision-support information, not as a substitute for nursing assessment or physician judgment.

### 2.2 Study population, eligibility criteria, and sampling

The study population comprised adults with type 2 diabetes who visited the endocrinology outpatient department of a tertiary-level Class A hospital. Eligible records met all of the following criteria: diagnosis of type 2 diabetes for at least 6 months; current glucose-lowering treatment; age 30–70 years; baseline glycated hemoglobin between 7.0 and 10.0%; ownership of a smartphone with basic operating ability; intact communication capacity; and sufficient baseline routine-care documentation to confirm eligibility and documented pathway exposure.

Records were excluded if patients had acute or severe chronic complications, cognitive impairment (Mini-Mental State Examination score <24) or a history of mental illness, participation in another clinical trial, pregnancy or lactation, or insufficient baseline documentation to confirm eligibility or pathway exposure. Completeness of week-24 outcomes was assessed after the baseline cohort had been defined and was not an initial eligibility criterion.

An *a priori* sample size calculation was used to estimate the minimum number of eligible cases required for this retrospective analysis, with glycated hemoglobin change designated as the primary outcome. The standard expression for comparing two independent means is presented as Equation 1. Parenthetical equation numbers are used only for cross-reference and are not bibliographic citations:

$$n=2\times {\left[\frac{(Z_{\alpha /2}+Z_{\beta })\times \sigma }{\delta }\right]}^{2}$$ (1)

In Equation 1, n is the required sample size per pathway, Zα/2 is the two-sided critical value (1.96), Zβ is 0.84 for 80% power, σ is the assumed standard deviation (0.8%), and δ is the clinically meaningful between-pathway difference in glycated hemoglobin change (0.5%). Substitution of these values yielded *n* = 40.14, which was rounded up to 41 records per pathway. Inflating this requirement by 15% for potentially incomplete 24-week outcomes produced a target of 49 records per pathway (41/0.85 = 48.24, rounded up). All 120 eligible records available during the predefined 12-month study period were included, exceeding the minimum target. Records were reviewed consecutively and classified according to documented routine-care pathway exposure; no pathway was assigned for research purposes. Sixty records were classified in each pathway at baseline. Complete 24-week outcomes were available for 57 patients in the nurse-led pathway and 56 in usual care, yielding an available-case outcome cohort of 113 and an incomplete-outcome rate of 5.8% (7/120). Baseline characteristics are reported for all 120 eligible records, whereas outcome analyses use the 113 complete 24-week records.

**Figure 1:**
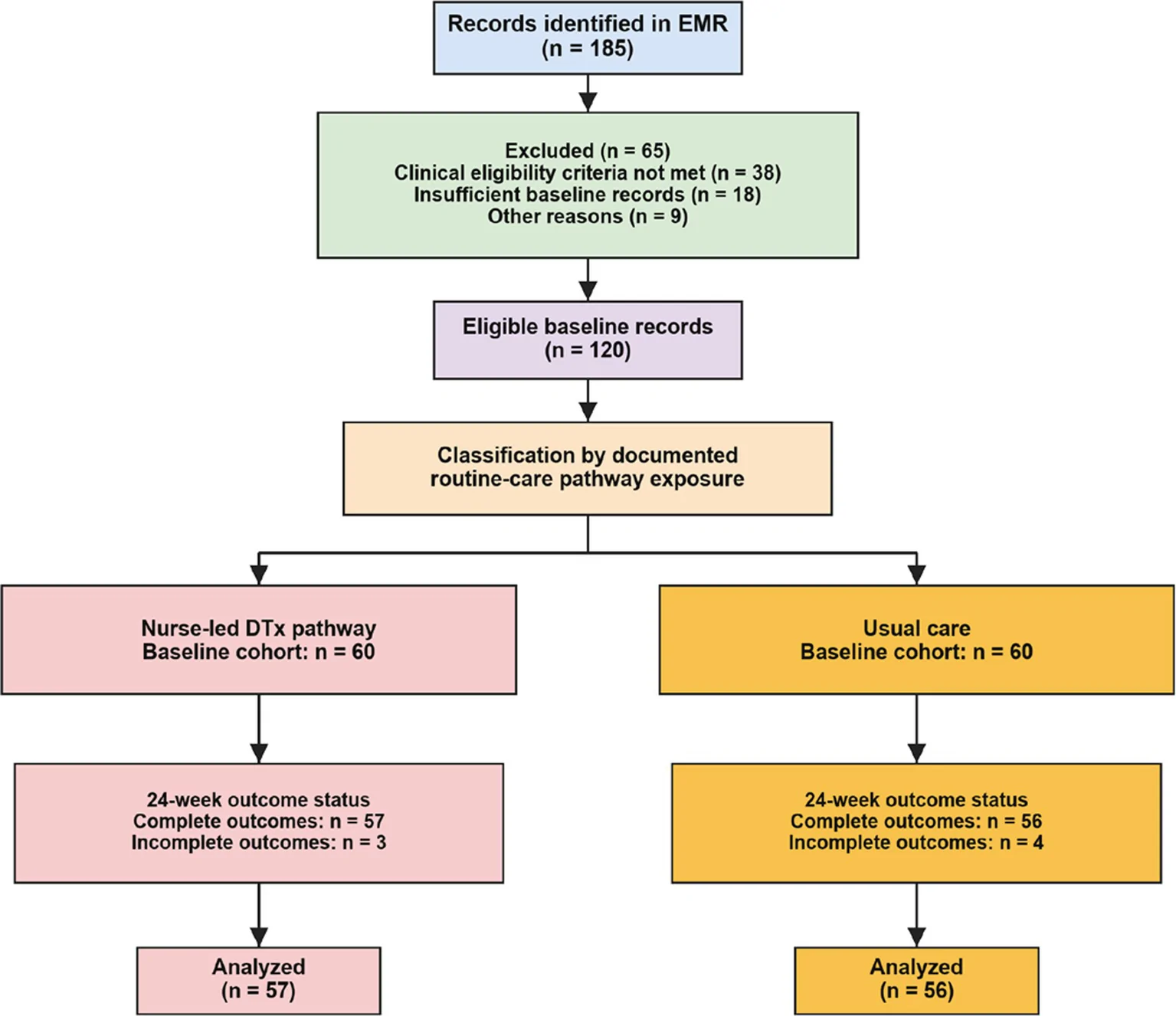
Data selection flow diagram for the retrospective cohort study.

The screening and record-selection process is shown in Figure 1.

Figure 1 presents record identification, baseline eligibility, classification by documented pathway exposure, and the final available-case outcome population. Initially, 185 electronic medical records were identified. Sixty-five were excluded because clinical eligibility criteria were not met (*n* = 38), baseline documentation was insufficient (*n* = 18), or other exclusion criteria applied (*n* = 9), leaving 120 eligible baseline records. All eligible records came from the same predefined 12-month period and were classified according to the routine-care pathway documented in the record: 60 in the nurse-led DTx pathway and 60 in usual care. This was retrospective classification, not research assignment. At week 24, complete outcomes were available for 57 and 56 patients, respectively; seven records with incomplete 24-week outcomes were excluded from outcome analyses.

### 2.3 Definition of comparison pathways and care components

The digital tool used in routine care was a hospital-adopted diabetes self-management mobile application. It enabled blood glucose logging, medication reminders, secure messaging, and nurse access to a backend dashboard for adherence monitoring. The application did not generate algorithm-driven therapeutic recommendations; it functioned as a data-capture, communication, and follow-up support tool. Medication adjustment decisions remained the responsibility of treating physicians in both pathways under the same hospital diabetes treatment protocol.

#### 2.3.1 Nurse-led digital therapeutics pathway

The nurse-led digital therapeutics pathway was organized into four stages: baseline assessment (week 1), initiation (weeks 2–4), maintenance (weeks 5–20), and evaluation (weeks 21–24). Routine records indicated that nurses delivered a closed-loop process of assessment, matching, education, monitoring, feedback, and escalation. The pathway connected mobile health tools with clinical judgment and was intended to reduce unsupported or inconsistent use of the technology.

Baseline assessment (week 1): Nurses assessed suitability for the digital therapeutics pathway across four dimensions: digital health literacy, disease awareness, self-efficacy, and technology acceptance (14, 15). The assessment was completed during one 30-min face-to-face outpatient visit using a standardized paper checklist developed by the hospital’s diabetes nursing committee. The locally developed operational score is presented as Equation 2:

$$S=0.30\times X_{1}+0.25\times X_{2}+0.25\times X_{3}+0.20\times X_{4}$$ (2)

In Equation 2, S represents the comprehensive applicability score, and X1-X4 are the standardized scores for digital health literacy, disease awareness, self-efficacy, and technology acceptance, respectively. Patients were categorized as having high applicability (S ≥ 70 points), moderate applicability (50 ≤ S < 70 points), or low applicability (S < 50 points). These cutoffs were derived from clinical experience and a pilot test involving 15 patients (data not shown), because no validated digital-readiness score specific to digital therapeutics use in type 2 diabetes was available. The score should therefore be regarded as an exploratory clinical-feasibility tool rather than a definitive classification instrument.

Initiation phase (weeks 2–4): Nurses provided two 45-min individual education sessions and one 30-min reinforcement session. Sessions were delivered face-to-face in the outpatient clinic and followed demonstration, guided practice, and feedback. All patients received the same 12-page illustrated booklet covering DTx operation, blood glucose management, lifestyle advice, and medication-related reminders. Patients with low digital confidence received an additional 15-min telephone call after each session.

Maintenance phase (weeks 5–20): Nurses monitored adherence through the backend dashboard and routine follow-up records. The score incorporated frequency of use, functional coverage, data completeness, and duration of use, drawing on the literature on technology-enabled diabetes care and adherence monitoring (24, 25). The locally used adherence score is presented as Equation 3:

$$C=0.30\times \frac{F}{7}+0.25\times \frac{M}{N}+0.25\times R+0.20\times \frac{T}{T_{0}}$$ (3)

In Equation 3, C represents the adherence score; F, M, R, and T represent login frequency, functional coverage, blood glucose record completeness, and duration of use, respectively. Three warning levels were defined from this score. Green (C ≥ 0.70) prompted a routine follow-up call every 2 weeks (10–15 min, focused on encouragement and troubleshooting). Yellow (0.50 ≤ C < 0.70) prompted weekly calls (15–20 min) and nurse review of the preceding week’s glucose logs; if no improvement was documented after 2 weeks, care was escalated to a face-to-face visit. Red (C < 0.50) prompted intensive follow-up within 48 h, including a clinic visit, re-education with the same standardized materials, and joint physician review to consider treatment adjustment. These actions were documented in the hospital’s digital therapeutics nursing protocol and applied throughout the study period. The 0.70 and 0.50 cutoffs were selected through clinical judgment and consensus among three senior diabetes nurses; they remain preliminary and require future validation.

Evaluation phase (weeks 21–24): Feedback reports were generated every 4 weeks throughout the pathway (weeks 4, 8, 12, 16, 20, and 24), with the final report forming part of the evaluation phase. Each report required approximately 20 min to prepare and was discussed during a 15-min face-to-face or telephone consultation. The reports supported shared decision-making among physicians, nurses, and patients and helped convert digital records into clinically meaningful care discussions.

#### 2.3.2 Usual care pathway

Patients receiving usual care underwent standard outpatient diabetes management under the same hospital treatment protocol. At enrollment or routine clinic contact, they received brief face-to-face guidance on downloading and installing the same mobile application, together with a printed user manual. Follow-up occurred mainly through routine outpatient visits approximately every 4 weeks, when physicians reviewed clinical status and any glucose records voluntarily presented by patients. Nurses could answer practical questions during routine clinic flow, but usual care did not include scheduled digital readiness assessment, structured onboarding sessions, dashboard-based adherence monitoring, tiered alert-triggered follow-up, four-weekly personalized feedback reports, or systematic nurse-physician-technical team coordination. Telephone, WeChat, and in-app follow-up were not delivered according to a predefined usual-care protocol. Routine records documented the brief installation guidance but did not systematically capture task-level nursing time, professional contact duration, unit costs, or full resource use for usual care. Workload findings were therefore limited to a descriptive estimate for implementing the nurse-led pathway; no comparative resource-use or economic analysis was undertaken.

**Figure 2:**
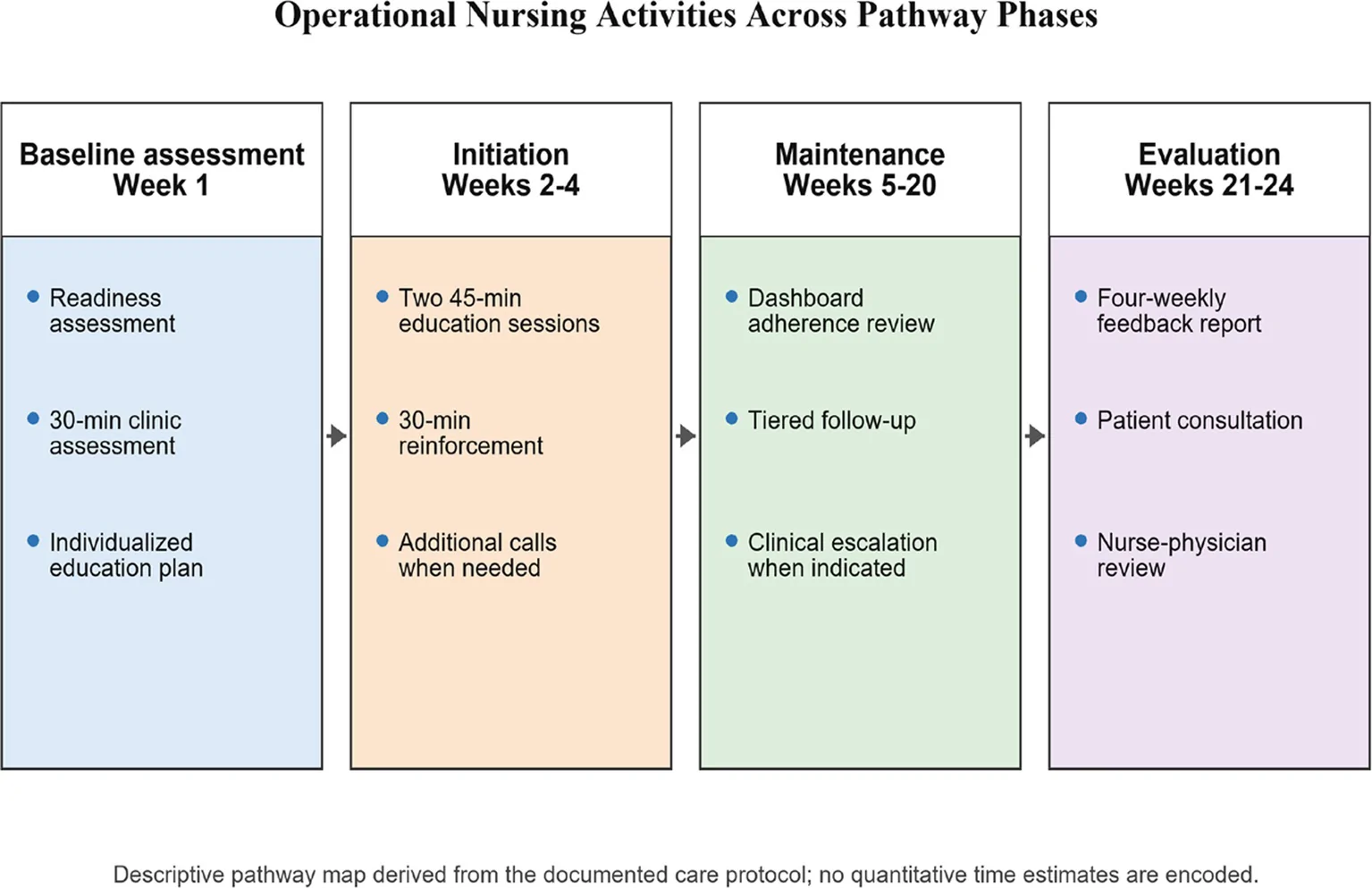
Operational nursing activities across phases of the nurse-led DTx pathway.

**Figure 3:**
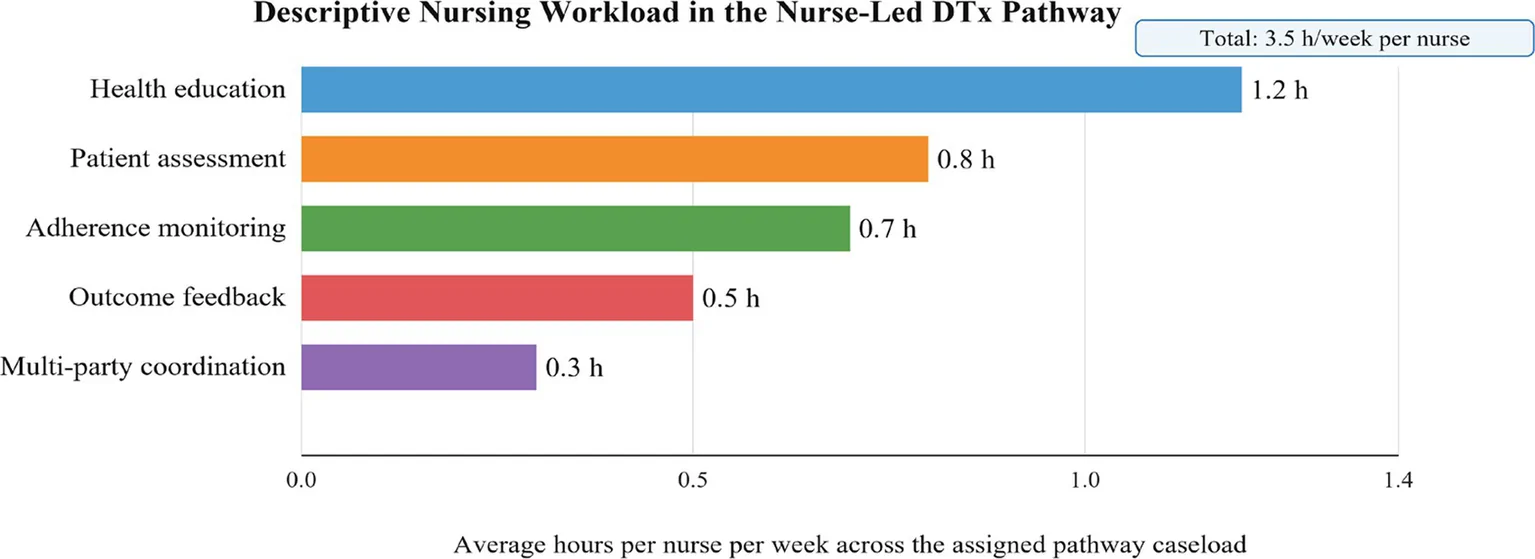
Descriptive nursing workload in the nurse-led digital therapeutics pathway.

Figure 2 provides a qualitative map of the operational activities documented across the four pathway phases. It does not encode hours or other quantitative workload estimates. Baseline assessment focused on readiness and care planning; initiation emphasized education and reinforcement; maintenance centered on dashboard review, tiered follow-up, and escalation; and evaluation incorporated feedback reports and clinical review. Quantitative workload is reported separately in Figure 3 using a single denominator: average hours per nurse per week across the assigned pathway caseload.

### 2.4 Professionals delivering the pathway and interprofessional coordination

The nurse-led DTx pathway involved structured collaboration among physicians, nurses, patients, and the technical team. Nurses served as the central information hub: they summarized weekly digital and clinical data for physicians, maintained patient contact through outpatient follow-up, telephone calls, WeChat groups, and in-app messaging, and transmitted usability or safety-related technical issues to the technical team. All nurses involved in the pathway (*n* = 4) were certified diabetes nurses with at least 3 years of experience. Before the study period, they completed a standardized 4-h workshop covering the readiness checklist, backend dashboard use, adherence-warning interpretation, documentation procedures, and communication for yellow/red alerts. A 12-page nursing manual was available on the hospital intranet. No additional training or staff support was introduced for research purposes.

**Figure 4:**
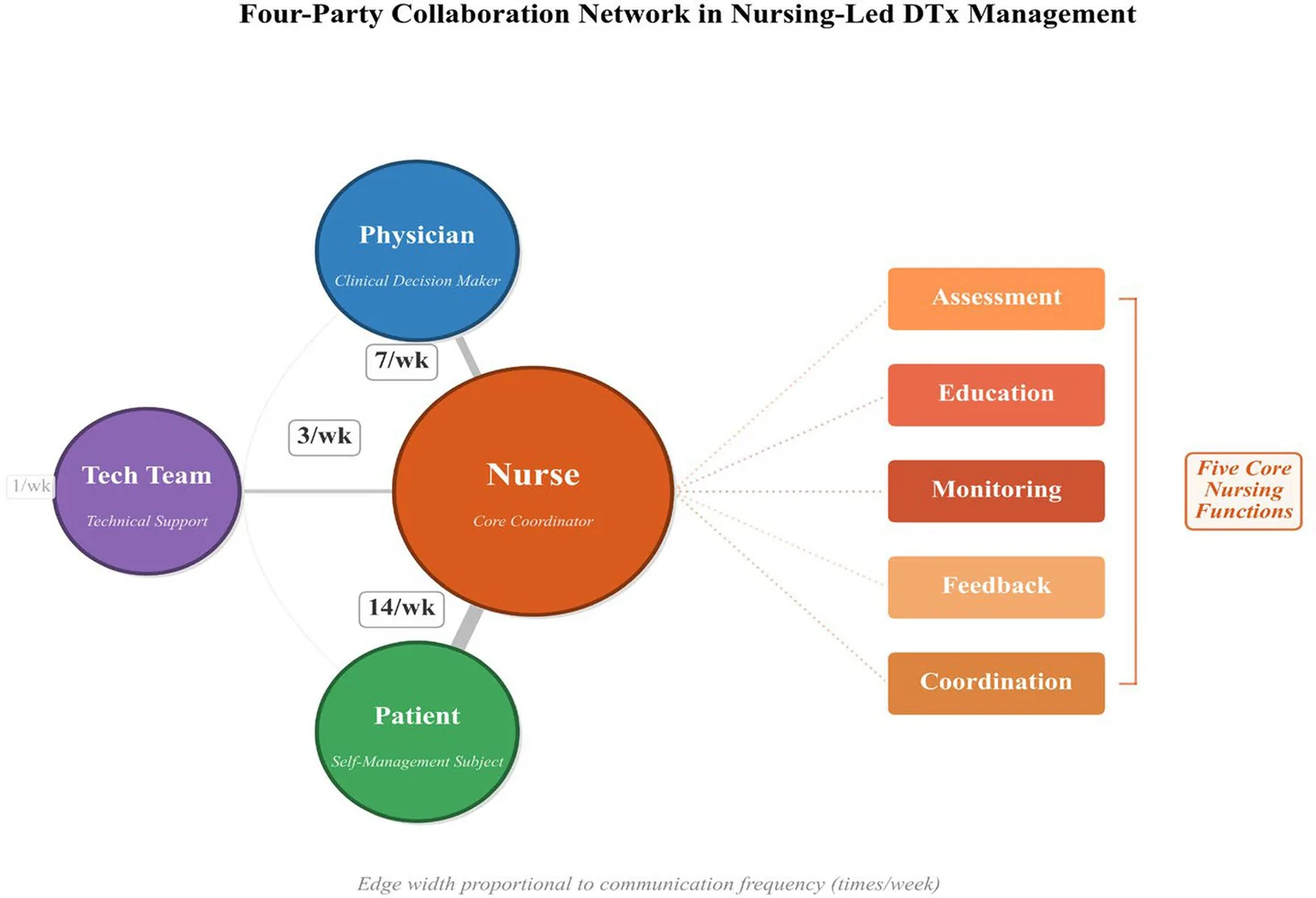
Four-party collaboration among physicians, nurses, patients, and the technical team.

The information flow path for the four-party collaboration is shown in Figure 4.

Figure 4 shows the nurse-centered collaboration network. Connection thickness indicates weekly communication frequency documented in routine records: nurse–patient interaction was most frequent, followed by nurse-physician, nurse-technical, and direct physician-patient communication. The figure illustrates how nurses bridged clinical decision-making, patient engagement, and technical troubleshooting. The five nursing functions—assessment, education, monitoring, feedback, and coordination—position nurses as digital care coordinators rather than one-time application instructors.

### 2.5 Variables, data collection, and outcome measures

Primary outcomes were DTx adherence and glycated hemoglobin change. These indicators captured behavioral continuity and glycemic change, both of which are central to evaluating digital chronic disease care in routine practice.

Secondary outcomes included patient satisfaction, self-management behavior, and the coefficient of variation of blood glucose. Patient satisfaction was assessed by the PSQ-18, self-management behavior by the Summary of Diabetes Self-Care Activities, and glycemic variability by continuous glucose monitoring data (26–29). Glycated hemoglobin change was calculated as the week-24 value minus the baseline value, with negative values indicating improvement. DTx adherence was calculated as the proportion of prescribed days with documented application use over the 168-day observation period. The coefficient of variation was derived from continuous glucose monitoring data as the standard deviation divided by the mean glucose level and expressed as a percentage.

### 2.6 Statistical analysis

Statistical analysis was performed using SPSS 26.0. Continuous variables were summarized as means and standard deviations or as medians and interquartile ranges depending on distributional characteristics. Categorical variables were summarized as frequencies and percentages. Normality was assessed using the Shapiro–Wilk test and Q-Q plots before parametric testing. Between-pathway comparisons were conducted using independent-samples t-tests where assumptions were met and non-parametric alternatives where appropriate. Categorical variables were compared using chi-square or Fisher’s exact tests. Effect sizes and 95% confidence intervals were reported to support interpretation beyond statistical significance.

Multiple linear regression was used to explore factors associated with endpoint DTx adherence. Predictor variables were selected *a priori* based on clinical plausibility and previous literature on digital engagement and diabetes self-management: pathway exposure, baseline eHEALS score, age, disease duration, education level, and baseline glycated hemoglobin (14, 15, 18). Model assumptions were assessed through partial regression plots, residual diagnostics, the Durbin-Watson statistic, the Breusch-Pagan test, and variance inflation factors. The Durbin-Watson statistic was 1.92, the Breusch-Pagan test was not significant (*p* = 0.31), residuals were approximately normal (Shapiro–Wilk *p* = 0.18), VIF values ranged from 1.12 to 1.38, and all tolerance values were above 0.70. Outcome analyses used the 113 records with complete 24-week outcomes, and no outcome values were imputed. Regression analyses used complete records for the prespecified variables within this outcome cohort.

To reduce potential confounding due to non-randomized pathway exposure, the regression model included six clinically relevant covariates. Propensity score matching was not performed because the modest sample size was insufficient to support stable matching without substantial case loss. Sensitivity analysis using entropy balancing yielded similar estimates, suggesting that the observed associations were robust within the limits of available variables. Statistical significance was set at *p* < 0.05.

Antidiabetic medication adjustments, insulin use, diet, exercise, comorbidities, and full resource-utilization indicators were not systematically recorded in the available retrospective data. All patients received care from the same medical team following the same hospital treatment protocol, and these unmeasured factors were considered when interpreting the observational findings.

### 2.7 Ethical considerations

Because this study was a retrospective analysis of de-identified data from routine clinical care, it involved no additional intervention, no direct patient interaction, and no collection of new identifiable private information. According to the Measures for Ethical Review of Life Science and Medical Research Involving Human Subjects (30), such research is exempt from informed consent and specific ethical approval. The Institutional Review Board of the participating hospital confirmed the exempt status (exemption notification on file). All data were anonymized before analysis to protect privacy and confidentiality in digital health monitoring.

## 3 Results

### 3.1 Baseline characteristics

Baseline demographic and clinical characteristics of patients in the two pathways are presented in Table 1. The two cohorts were broadly comparable at baseline on the variables available in the retrospective records.

**Table 1:** Baseline characteristics of the study population.

Characteristic	Nurse-led DTx pathway (baseline *n* = 60; outcome *n* = 57)	Usual care (baseline *n* = 60; outcome *n* = 56)	*p*-value
Age (years), mean ± SD	52.3 ± 9.8	53.1 ± 10.2	0.672
Male, *n* (%)	32 (53.3)	29 (48.3)	0.583
Disease duration (years), mean ± SD	6.2 ± 3.5	5.8 ± 3.2	0.523
Baseline HbA1c (%), mean ± SD	8.1 ± 0.8	8.0 ± 0.9	0.528
Body mass index (kg/m^2^), mean ± SD	25.8 ± 3.2	26.1 ± 3.4	0.617
Education level, *n* (%)			0.724
Primary school	8 (13.3)	10 (16.7)	
Middle school	22 (36.7)	20 (33.3)	
High school	18 (30.0)	19 (31.7)	
College or above	12 (20.0)	11 (18.3)	
eHEALS score, mean ± SD	28.5 ± 5.2	27.9 ± 5.6	0.543

All data were extracted from electronic medical records. Continuous variables were compared using independent-samples *t*-tests, and categorical variables were compared using chi-square or Fisher’s exact tests, as appropriate. Data are presented as mean ± standard deviation or n (%). Baseline characteristics are shown for all 120 eligible records. Outcome analyses used records with complete 24-week outcomes (nurse-led DTx pathway *n* = 57; usual care *n* = 56).

### 3.2 Primary outcomes

DTx adherence and glycated hemoglobin change are summarized in Table 2. Between-pathway differences were reported with 95% confidence intervals to indicate the precision of the estimates.

**Table 2:** Digital therapeutics adherence and glycated hemoglobin change by pathway.

Outcome	Nurse-led DTx pathway (*n* = 57)	Usual care (*n* = 56)	Difference (95% CI)	*p*-value
Digital therapeutics adherence rate (%)	78.3 ± 12.5	54.7 ± 18.2	23.6 (17.2–30.0)	<0.001
Glycated hemoglobin change (%)	−0.8 ± 0.4	−0.3 ± 0.5	−0.5 (−0.7 to −0.3)	<0.001

As shown in Table 2, adherence was higher in the nurse-led DTx pathway than in usual care, with a between-pathway difference of 23.6 percentage points (78.3% vs. 54.7%, *p* < 0.001). Glycated hemoglobin reduction was also larger in the nurse-led DTx pathway, with a difference of 0.5 percentage points (−0.8% vs. −0.3%, p < 0.001). This association is clinically plausible because onboarding education may have reduced operational barriers to app use, while tiered follow-up allowed nurses to identify declining engagement early. Usual care also showed modest glycemic improvement, suggesting that the digital tool itself may have some value; however, the observed gains were stronger when the tool was embedded in a structured nursing continuity pathway.

**Figure 5:**
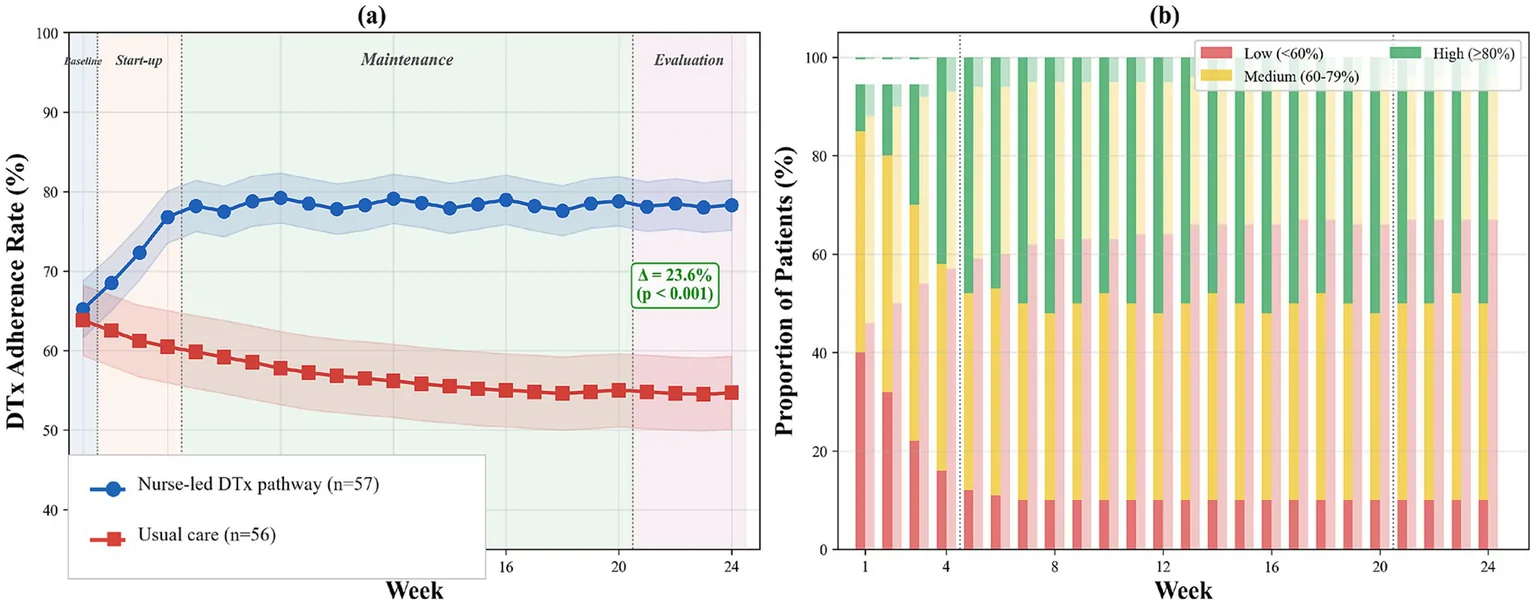
Digital therapeutics adherence over 24 weeks. **(a)** Weekly mean adherence rates for the nurse-led DTx pathway (*n* = 57) and usual care (*n* = 56), with shaded 95% confidence intervals. **(b)** Weekly distributions of low (<60%), medium (60–79%), and high (≥80%) adherence within the same complete 24-week outcome cohort. At each week, the left stacked bar represents the nurse-led DTx pathway, and the right stacked bar represents usual care.

To examine adherence over time, weekly behavioral data were extracted from clinical documentation and backend usage records for the 113 patients with complete 24-week outcomes. No weekly adherence values were imputed. The results are shown in Figure 5.

Figure 5a shows study weeks 1–24 on the horizontal axis and adherence on the vertical axis for the complete 24-week outcome cohort (nurse-led DTx pathway *n* = 57; usual care *n* = 56). The two pathways had similar adherence at baseline, but their trajectories diverged after initiation. Adherence in the nurse-led DTx pathway rose rapidly to approximately 77%, remained stable during maintenance, and ended at 78.3%. In usual care, adherence gradually declined to 54.7%, widening the between-pathway gap to 23.6 percentage points (*p* < 0.001). Figure 5b shows the distribution of adherence categories over time within the same complete-case cohort. These findings indicate an association between structured nursing support and both the mean level and distribution of adherence.

**Figure 6:**
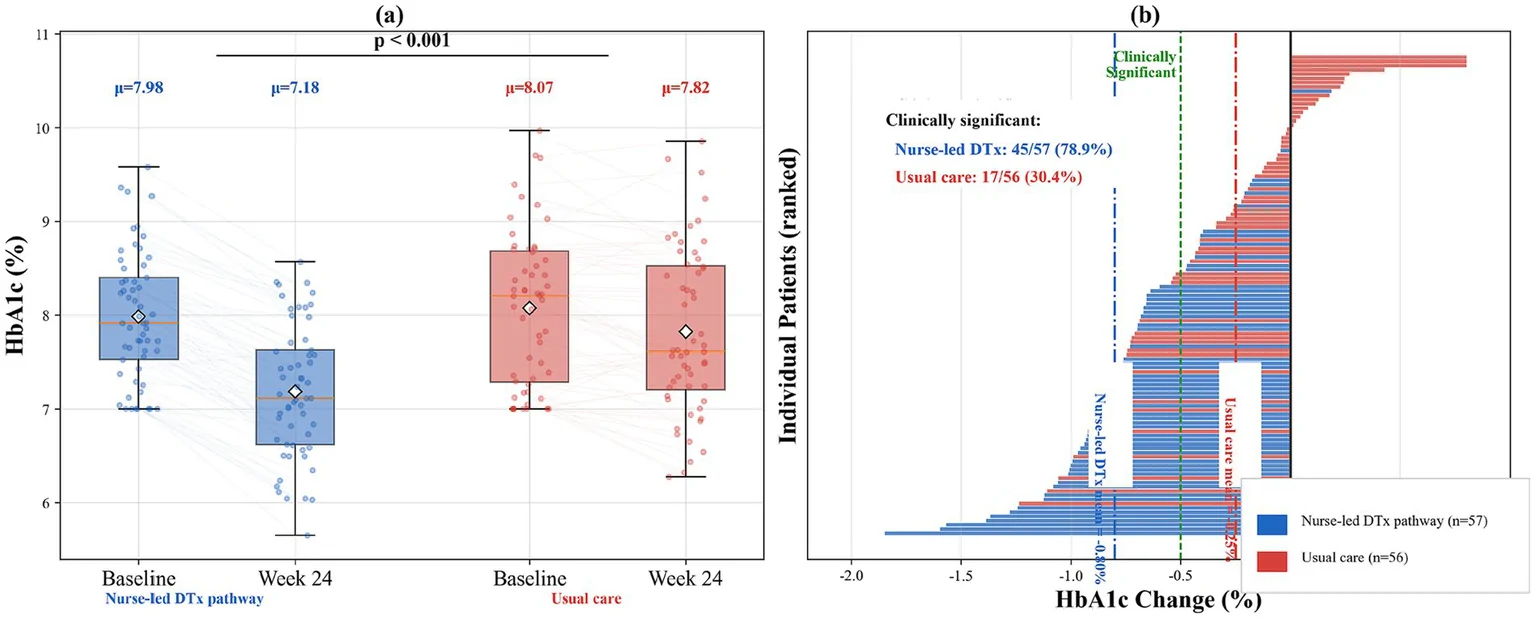
Distribution of glycated hemoglobin change by pathway. **(a)** HbA1c distribution: baseline vs. endpoint. **(b)** Individual HbA1c change waterfall plot.

Glycated hemoglobin changes were analyzed from group-level and individual perspectives, as shown in Figure 6.

Figure 6a shows baseline and week-24 glycated hemoglobin values. Values decreased from 7.98 to 7.18% in the nurse-led DTx pathway and from 8.07 to 7.82% in usual care. The between-pathway difference was statistically significant (p < 0.001). Paired trajectories show that most patients in the nurse-led DTx pathway improved in the same direction, whereas usual care showed greater variability. Figure 6b confirms this pattern: 78.9% of patients in the nurse-led DTx pathway achieved a clinically meaningful reduction of at least 0.5%, compared with 30.4% in usual care. These findings suggest that nursing support may reduce heterogeneity in patient response by helping patients use digital functions more consistently.

### 3.3 Secondary outcomes

Secondary outcomes included patient satisfaction, self-management behavior, and glycemic variability. Three indicators were analyzed: PSQ-18 score, change in Summary of Diabetes Self-Care Activities score, and change in blood glucose coefficient of variation. Results are shown in Table 3.

**Table 3:** Satisfaction, self-management behavior, and glycemic variability by pathway.

Outcome	Nurse-led DTx pathway (*n* = 57)	Usual care (*n* = 56)	Difference (95% CI)	*p*-value
PSQ-18 score	80.3 ± 8.2	68.5 ± 11.3	11.8 (7.9–15.7)	<0.001
SDSCA change (score)	+1.8 ± 0.9	+0.6 ± 0.7	1.2 (0.9–1.5)	<0.001
CV change (%)	−8.7 ± 4.2	−3.6 ± 3.8	−5.1 (−6.5 to −3.7)	<0.001

Table 3 shows that each retained secondary outcome favored the nurse-led DTx pathway. The pathway was associated with a higher PSQ-18 score, greater improvement in self-management behavior, and a larger reduction in glycemic variability. These coordinated changes suggest that digital therapeutics use was more sustained when nurses helped translate application functions into everyday routines.

The pattern of secondary outcomes supports a nursing interpretation rather than a purely technical one. Higher satisfaction was plausibly related to timely responses, individualized communication, and sustained guidance. The larger improvement in self-management behavior suggests that nurses helped patients convert recording, reminder, and feedback functions into repeated daily practice. The reduction in glycemic variability is also clinically relevant because it reflects more stable metabolic control in addition to lower average glycated hemoglobin. Together, these results suggest multidimensional associations across patient experience, behavior, and physiological stability.

To clarify improvement patterns, self-management behavior was analyzed across five SDSCA dimensions: glucose monitoring, special diet, general diet, exercise, and foot care. Standardized effect sizes were calculated for each dimension to assess practical significance. Patient satisfaction was further examined across seven PSQ-18 dimensions using a radar chart, allowing identification of the service domains most associated with the nurse-led DTx pathway.

**Figure 7:**
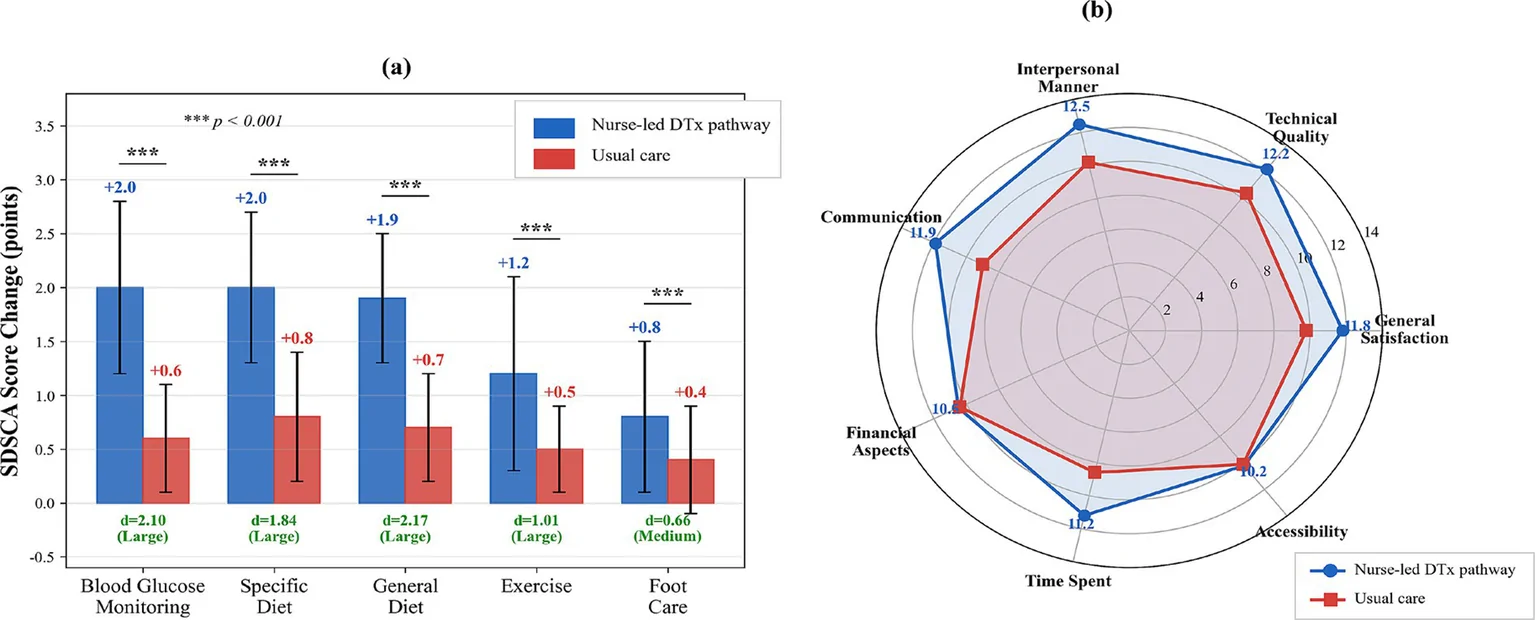
Multidimensional comparison of satisfaction and self-management behavior by pathway. **(a)** Self-management behavior improvement. **(b)** PSQ-18 satisfaction radar chart.

Figure 7a shows that patients in the nurse-led DTx pathway had greater observed improvement than those receiving usual care in all five self-care dimensions. The largest difference appeared in glucose monitoring, followed by special diet and general diet. This pattern is consistent with the application’s strengths in blood glucose recording and dietary prompting, while nurses helped convert these functions into stable habits. Smaller differences appeared in exercise and foot care, suggesting that these behaviors may require additional offline reinforcement. Figure 7b shows higher satisfaction scores across all dimensions, with the largest differences in communication and consultation time.

### 3.4 Factors associated with digital therapeutics adherence

Multiple linear regression was used to examine factors associated with endpoint DTx adherence. The dependent variable was endpoint adherence, and six clinically relevant predictors were included: pathway exposure, baseline eHealth literacy score, age, disease duration, education level, and baseline glycated hemoglobin. Collinearity diagnostics were performed before regression analysis. The model fit was acceptable (R^2^ = 0.48, *p* < 0.001), indicating that the included variables explained nearly half of adherence variation.

**Figure 8:**
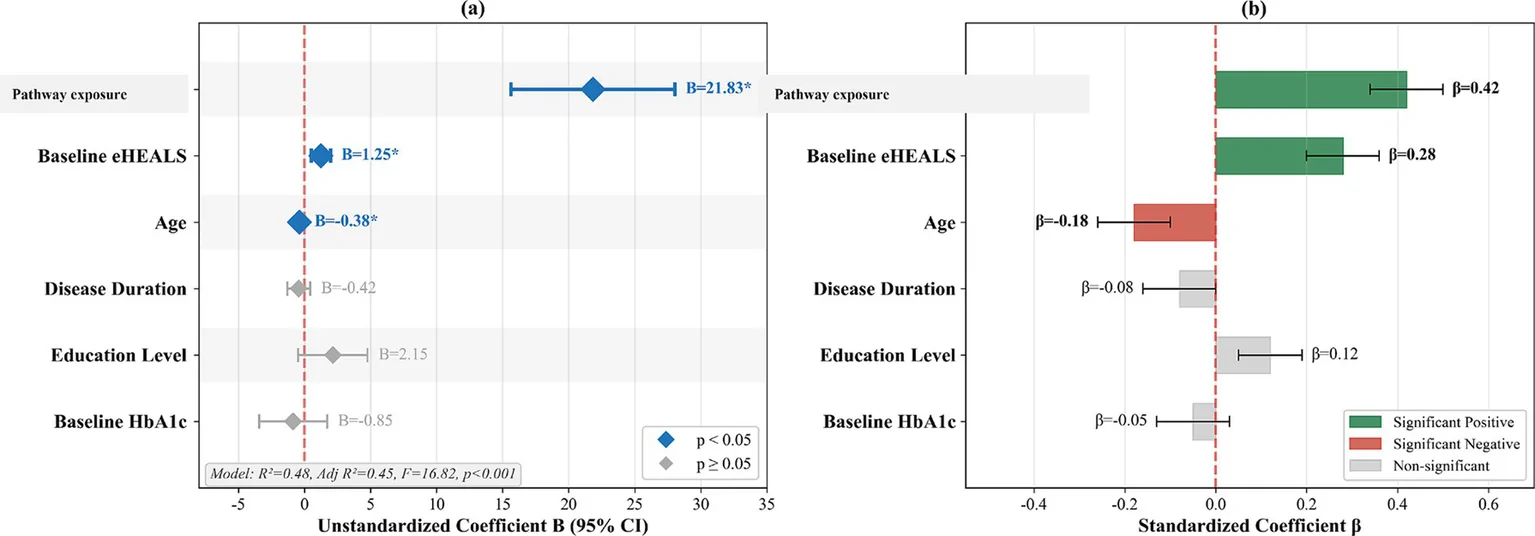
Forest plot of factors associated with digital therapeutics adherence. **(a)** Forest plot: regression coefficients. **(b)** Standardized coefficients (VIF all <1.4).

Figure 8a reports unstandardized regression coefficients and 95% confidence intervals. Three variables were statistically significant: pathway exposure showed the strongest positive association with adherence (B = 21.83, *p* < 0.05), baseline eHealth literacy was also positively associated with adherence (B = 1.25, p < 0.05), and age was negatively associated with adherence (B = -0.38, p < 0.05). Disease duration, education level, and baseline glycated hemoglobin were not significant adherence predictors. Figure 8b shows relative importance using standardized coefficients, with pathway exposure and baseline eHealth literacy as the most important positive predictors. Variance inflation factor values were below 1.4, suggesting no meaningful collinearity.

### 3.5 Descriptive nursing workload

Implementation sustainability depends on workload and resource requirements as well as clinical outcomes. Available nurse work logs were used to describe DTx-related nursing time for the nurse-led pathway as average hours per nurse per week across the assigned pathway caseload. Equivalent task-level time allocation, unit-cost information, and full resource-use data for usual care were not systematically captured. Consequently, the analysis was restricted to descriptive nursing workload for the nurse-led pathway; pathway-specific cost estimates and comparative economic indicators were not reported.

Figure 3 shows the distribution of average DTx-related nursing time, expressed as hours per nurse per week across the assigned nurse-led pathway caseload. The five task categories sum to 3.5 h per nurse per week: health education 1.2 h, patient assessment 0.8 h, adherence monitoring 0.7 h, outcome feedback 0.5 h, and multi-party coordination 0.3 h. Because equivalent task-level time and verifiable unit-cost data for usual care were unavailable, no pathway-specific cost comparison or cost-effectiveness estimate is presented.

## 4 Discussion

In this retrospective cohort, the nurse-led digital therapeutics pathway was associated with higher adherence, greater glycated hemoglobin reduction, better patient satisfaction, improved self-management behavior, and lower glycemic variability than usual care. These results are observational associations, not proof of causal effectiveness. Their direction is nevertheless clinically plausible and consistent with evidence that digital diabetes tools perform best when embedded in structured care rather than offered as stand-alone applications (8–10). Pathway exposure showed the strongest adjusted association with adherence, which points to onboarding, monitoring, feedback, and escalation as plausible mechanisms through which nurses may help patients sustain digital self-management.

Viewed alongside earlier research, the present results accord with randomized and systematic-review evidence that mobile and digital diabetes interventions can improve glycated hemoglobin, while engagement and implementation quality strongly shape outcomes (8, 11, 31). They are also compatible with real-world findings from combined digital health and glucose-monitoring programs (32). Unlike studies centered on stand-alone applications, this analysis suggests that a nurse-led pathway may help maintain adherence by reducing the practical burden of app use, translating recorded data into self-care actions, and identifying disengagement before it becomes persistent. This interpretation extends diabetes self-management education literature, which identifies ongoing professional support, patient-centered problem solving, and structured follow-up as central elements of effective care (18, 20, 21).

The study’s contribution lies less in the application itself than in the organization of nursing work around it. Readiness assessment, individualized onboarding, dashboard-based adherence monitoring, warning-level follow-up, periodic feedback, and coordination among patients, nurses, physicians, and technical staff formed a single care pathway. This configuration is consistent with implementation-science frameworks that distinguish an intervention’s components from the systems required to sustain them in routine practice (12, 33, 34). It also reflects complex intervention guidance, in which outcomes depend on context, delivery, adaptation, and interaction with users as well as on intervention content (35, 36). By specifying these nursing activities, the analysis contributes to the practical discussion of digitally enabled nursing and continuity in chronic disease care.

This pathway-level interpretation has several clinical and patient-safety implications. Patients with lower digital readiness may need more than installation guidance; demonstration, practice, reassurance, and a clear route to assistance may be needed when data entry or interpretation becomes difficult (14, 15). Nurse review of adherence and glucose records may also reduce the likelihood that app fatigue, incomplete monitoring, or abnormal glucose patterns go unnoticed between clinic visits. Defined escalation rules help preserve professional boundaries: the digital tool supports self-management and communication, while nurses and physicians retain responsibility for clinical interpretation and treatment decisions. Such boundaries matter in diabetes care because glycemic variability and time-in-range indicators are informative only when interpreted in clinical context (26, 28).

Implementation also requires a cautious reading of workload and resource findings. Available nurse work logs indicated that education, assessment, adherence monitoring, feedback, and coordination together required an average of 3.5 h per nurse per week across the assigned pathway caseload. This descriptive workload may inform staffing discussions, but it cannot establish comparative efficiency, affordability, or cost-effectiveness. Equivalent task-level nursing time, verifiable unit costs, and complete resource-use data for usual care were not systematically captured; therefore, no pathway-specific cost estimates or economic comparisons are reported. Prospective implementation research should measure professional time, technical support, medication changes, outpatient visits, adverse events, and patient-incurred costs in both pathways before conclusions about scalability or economic value are drawn.

The findings should be considered in light of several limitations. The retrospective, single-center design precludes causal inference and limits external validity. Classification by documented pathway exposure may have introduced selection bias and residual confounding, even though recorded baseline variables were broadly comparable and sensitivity analysis supported the direction of the estimates. The outcome cohort was modest and did not support a reproducible interaction analysis; no subgroup inference is therefore presented. Medication adjustments, insulin use, comorbidities, diet, exercise, and some social determinants of digital access were not systematically recorded. The readiness score and adherence-warning thresholds were developed locally and require external validation. In addition, usual-care time allocation, professional involvement, and complete resource utilization were incompletely documented, preventing a full comparative economic analysis. Generalizability may also be limited in institutions with different staffing models, digital platforms, patient populations, or reimbursement structures.

Prospective work should therefore evaluate nurse-led digital therapeutics pathways across multiple centers, use validated measures of digital readiness and eHealth literacy, predefine patient-safety outcomes, and collect comparable resource-use data for both pathways. A pragmatic trial or prospective implementation study could also examine treatment adjustments, engagement trajectories, and differential outcomes among older adults, patients with lower digital literacy, and those with poorer baseline glycemic control.

## 5 Conclusion

In this single-center retrospective cohort, the nurse-led digital therapeutics pathway was associated over 24 weeks with higher adherence and more favorable glycemic, self-management, and patient-experience outcomes than usual care. Pathway exposure also showed the strongest adjusted association with adherence. These observations do not establish causality, but they support the clinical plausibility of linking digital tools with readiness assessment, patient education, adherence monitoring, feedback, safety escalation, and continuity of care. Prospective multicenter studies using validated readiness measures, systematic workload data for both pathways, and formal economic methods are needed before broader implementation claims can be justified.

## Data Availability

The original contributions presented in the study are included in the article/supplementary material, further inquiries can be directed to the corresponding author.
